# Evaluation of the Technical Viability of Distributed Mechanical Recycling of PLA 3D Printing Wastes

**DOI:** 10.3390/polym13081247

**Published:** 2021-04-12

**Authors:** Freddys R. Beltrán, Marina P. Arrieta, Eduardo Moreno, Gerald Gaspar, Luisa M. Muneta, Ruth Carrasco-Gallego, Susana Yáñez, David Hidalgo-Carvajal, María U. de la Orden, Joaquín Martínez Urreaga

**Affiliations:** 1 Escuela Técnica Superior de Ingenieros Industriales, Universidad Politécnica de Madrid, 28006 Madrid, Spain; f.beltran@upm.es (F.R.B.); eduardo.moreno.escribano@gmail.com (E.M.); geraldmanuel.gaspar@upm.es (G.G.); luisa.mtzmuneta@upm.es (L.M.M.); ruth.carrasco@upm.es (R.C.-G.); susana.yanez@upm.es (S.Y.); david.hidalgo.carvajal@upm.es (D.H.-C.); joaquin.martinez@upm.es (J.M.U.); 2 Grupo de Investigación Polímeros Caracterización y Aplicaciones (POLCA), 28012 Madrid, Spain; mariula@ucm.es; 3 Facultad de Óptica y Optometría, Universidad Complutense de Madrid, 28037 Madrid, Spain

**Keywords:** poly (lactic acid), 3D printing, distributed recycling, mechanical recycling

## Abstract

3D printing PLA wastes were recovered from a well-known reference grade and from different sources. The recovered wastes were subjected to an energic washing step and then reprocessed into films by melt-extrusion, followed by compression molding to simulate the industrial processing conditions. The obtained materials were characterized and the optical, structural, thermal and crystallization behavior are reported. The mechanical recycling process leads to an increase of the crystallinity and a decrease of the intrinsic viscosity of the formulations, particularly in the sample based on blends of different 3D-PLA wastes. Moreover, the obtained films were disintegrated under composting conditions in less than one month and it was observed that recycled materials degrade somewhat faster than the starting 3D-PLA filament, as a consequence of the presence of shorter polymer chains. Finally, to increase the molecular weight of the recycled materials, the 3D-PLA wastes were submitted to a solid-state polymerization process at 110, 120, and 130 °C, observing that the recycled 3D-wastes materials based on a well-known reference grade experiences an improvement of the intrinsic viscosity, while that coming from different sources showed no significant changes. Thus, the results show that 3D printing PLA products provides an ideal environment for the implementation of distributed recycling program, in which wastes coming from well-known PLA grades can successfully be processed in films with good overall performance.

## 1. Introduction

3D printing is an additive manufacturing technique, which allows, in a layer-by-layer process, to obtain structures and geometries from 3D model data [1,2]. The simplicity, cheap cost and high customizability of 3D printing products are transforming the manufacturing and logistics processes, reaching several sectors such as construction, aerospace, automotive and biomedical industries [3,4,5]. The aforementioned advantages of 3D printing have become even more important during 2020 COVID-19 pandemic, in which the disruption in supply chains generated a shortage of critical goods in the fight against the pandemic [6]. The most commonly used polymers in 3D printing are thermoplastics such as acrylonitrile butadiene styrene (ABS), polycarbonate (PC), nylon, and poly (lactic acid) (PLA) [3,7]. Among all these polymers, PLA has gained a major interest for the development of 3D-printing products due to its overall balanced processability and performance [8], as well as due to its intrinsic sustainable characteristics since it is a biopolyester derived from renewable resources (i.e., sugar, corn, and beet) and it is also compostable [9]. For instance, coronamakers, a citizen maker’s movement having a 3D-printing machine that were associated during the initial period of COVID-19 pandemic, produced a lot of face masks, visors and parts for PPEs (personal protective equipment) based on 3D-printed PLA to kindly provide them to the society [10]. 

Despite the numerous advantages of 3D printing, it generates a considerable amount of plastic wastes (coming from disposable prototypes and rejected parts), which contributes to the global plastic waste contamination problem [11]. However, the 3D printing provides an ideal environment for the distributed recycling, in which each consumer directly recycles its own wastes [12,13]. In-home recycling of 3-D post-consumer plastic represents a novel path to a future of distributed recycling, since distributed recycling uses less embodied energy than its centralized recycling counterpart [14]. This approach would allow to reduce the environmental impact of 3D printing, and hence to improve the sustainability of the whole manufacturing process [15,16]. Therefore, it is expected that 3D printing networks will be rapidly expanding the potential of distributed recycling networks.

Although the environmental advantages of mechanical recycling are known, this process does not come without drawbacks. Previous studies, such as those conducted by Beltrán et al. [17], point out that mechanical recycling led to a decrease of the PLA molecular weight as it was shown by a reduction of the intrinsic viscosity along with some important properties of a PLA packaging grade. Some authors have evaluated the possibility of close-loop recycling in the 3D printing process. For instance, Cruz-Sanchez et al. [18] found that successive 3D printing cycles caused a slight improvement of the tensile modulus, along with a significant decrease of the tensile strength, both of which were related to the reduction of the molecular weight due to the degradation of the polymer during the printing process. In the same line of work, Anderson et al. found a slight decrease of the mechanical properties of 3D printed PLA samples, after one recycling process [19]. Zhao et al. [11] reported that two successive 3D printing cycles led to a decrease of the molecular weight, and an increase of the crystallization ability of PLA. Other authors, such as Pinho et al. [16] and Alexandre et al. [20], have evaluated the use of PLA wastes coming from different applications (such as food packaging and car parts) to produce 3D printed samples, reporting acceptable mechanical properties of the recycled PLA materials. However, PLA could not be infinitely recycled since each reprocessing cycle negatively influences the thermomechanical performance of PLA [8], due to a reduction of the molecular weight. This behavior limits the times that the material can be recovered in the form of recycled plastic. Fortunately, PLA is a compostable material [21] and, thus, recycled PLA-based products after its useful life can ultimately be composted closing the loop and being aligned with circular economy. 

The aforementioned studies provide useful information the contribution of 3D printing to the circular economy approach, by means of mechanical recycling. In fact, in a previous work the Material Circularity Indicator (MCI), an indicator of the degree of circularity developed by the Ellen McArthur Foundation [22], was calculated for 3D printed parts made with recycled PLA. The MCI showed a high level of circularity (MCI = 0.98) when new materials are printed from 100% recycled PLA [23]. However, from a technical point of view, factors such as the influence of heterogeneous origin of the recycled wastes, the influence of other processes presents in mechanical recycling, or the potential methods for improving the performance of the recycled materials have not been discussed. We have previously studied the technical viability of the mechanical recycling of 3D-printed PLA wastes [10], by analyzing the effect of a mechanical recycling process on the intrinsic viscosity. It was observed that the reduction on the intrinsic viscosity was higher when materials were prepared from blends of different PLA grades. 

Consequently, the main aim of the present research work is to thoroughly evaluate the mechanical recycling of 3D printing PLA wastes coming from different sources and to provide a scenario of the industrial recycling conditions, considering the presence of a demanding washing step prior to the reprocessing of the materials. Furthermore, an environmentally sound and cost-friendly upgrade method for the recycled materials is proposed. 

To achieve this, two residues were used: (i) 3D printing wastes from a well-known reference grade, printed under controlled conditions and (ii) a mixture of 3D printing wastes coming from different coronamakers agents in Spain. Both wastes were shredded, and then washed at conditions close to those used in plastics recycling industries. The washed samples were thoroughly dried and reprocessed by means of melt compounding into films. Then, the materials were melt compounded obtaining the well-known reference grade as well as a blend based on a mixture of the previously characterized materials. The structural characterization of the obtained materials was performed in each recovered material by means of FTIR spectroscopy. The solution viscometry was used as the main variable to determine the effect of 3D printing, washing, mechanical recycling and film forming processes on the viscosimetric molecular weight of the recycled materials. Differential scanning calorimetry (DSC), thermogravimetric analysis (TGA), colorimetry and nanoindentation tests were performed to obtain a complete characterization of the films and evaluate their industrial applicability. Moreover, the compostability of the films was also studied at laboratory scale level, by following the mass loss, thermal degradation, and the crystallinity during the composting test, with the aim to supply information about an environmentally friendly end-life option for the recycled materials since they cannot be recycled again. 

Finally, with the aim to extend PLA recycling, the resulting materials were subjected to a solid-state polymerization (SSP) process, by thermal treatments at 110, 120, and 130 °C, to improve their performance by increasing their molecular weight. The obtained results of recycled materials coming from both kind of 3D printed PLA wastes are discussed to get insights about the viability of distributed recycling. Results shows that SSP allowed to partially recover the average molecular weight of the recycled samples, although the heterogeneities present in the sample prepared from the mixture of 3D printing wastes led to a material with poorer properties. Meanwhile, the recycling of PLA 3-D waste coming from a homogeneous and well-known PLA grade leads to a recycled material with very good properties, especially when SSP processes are applied. These results allow to enhance the circularity of the 3-D printing with PLA and open new ways to develop simple and environmentally sound distributed recycling processes.

## 2. Materials and Methods

### 2.1. Materials

Two different PLA materials were analyzed in this study. The first material was a well-known 3D printing grade with a density of 1.24 g/mL, known as Smartfil^®^ PLA, commercialized by Smart Materials 3D. The second material was a blend formed by a mixture of four different 3D printing wastes, coming from an association of coronamakers (Madrid, Spain). The different wastes that were used to prepare the blend were a translucent PLA (25 %wt.), a green colored PLA (25 %wt.), a violet-colored PLA (25 %wt.) and white-colored (25 %wt.). A virgin PLA (Ingeo^TM^ 2003D, containing up to 4.1% isomeric D units), obtained under controlled conditions [17], is used as reference. 

### 2.2. 3D Printing Conditions of the Reference Grade

The reference grade was printed at room temperature using an ABAX PRI5 3D printer, operating at 200 °C and with a bed temperature of 50 °C.

### 2.3. Mechanical Recycling Process

**Figure 1:**
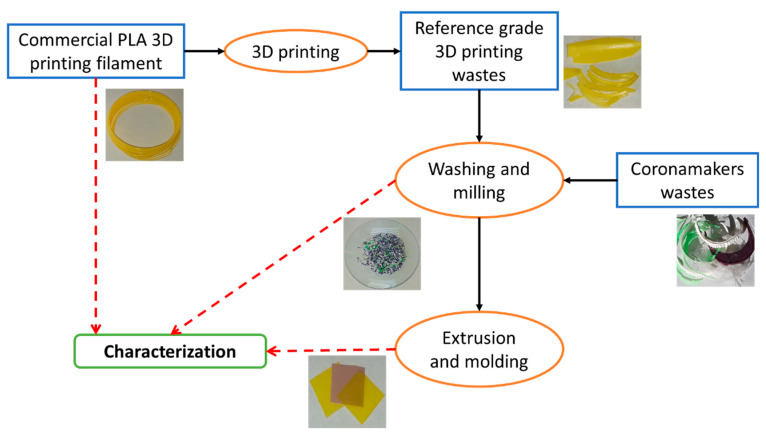
Scheme of the mechanical recycling process.

The mechanical recycling process is schematically represented in Figure 1. Both the printing wastes coming from the reference grade and the mixture coming from the coronamakers were grinded and subjected to a demanding washing process at 85 °C using an aqueous solution of NaOH (1.5 %wt.) and a surfactant (Triton X-100, 0.3 %wt.) which is similar to those used in the mechanical recycling industry [24]. Prior to processing, the washed materials were dried at 85 °C for 2 h in a vacuum oven. 

After drying, both materials were processed in a Rondol Microlab twin-screw extruder (L/D = 20), operating at 60 rpm, with following temperature profile (from hopper to die): 125, 160, 190, 190, and 180 °C. Then, the extruded samples were compression molded into films, with a thickness of 210 µm, using an IQAP-LAP at 190 °C. The pellets were kept between the hot-plates at atmospheric pressure for 4 min until melting. Then they were subjected to a degasification cycle of 1 min, followed by a pressure cycle at 14 MPa for 1 min. Finally, film samples were quenched between cold plates (at 14 MPa) to obtain transparent films. 

### 2.4. Thermal Treatments of the Recycled Samples

The mechanically recycled samples were subjected to thermal treatments at 110, 120, and 130 °C, in a vacuum oven for 8 h. Table 1 summarizes all the samples analyzed in this study.

### 2.5. Characterization Techniques

Fourier-transform infrared spectroscopy (FTIR) scans were recorded using a Nicolet iS10 spectrometer, equipped with an attenuated total reflectance (ATR) accessory. A resolution of 4 cm^−1^ and 16 scans were used. All the spectra were corrected and normalized at 1451 cm^−1^, an internal standard reported in the literature [25]. As it has been pointed out in previous works [26,27], FTIR spectroscopy can be used to calculate the variation of crystallinity of a sample, by means of Equation (1):

(1) $$X_{sample}=\left(\frac{I_{PLA-f}^{956}-I_{sample}^{956}}{I_{PLA-f}^{956}}\right)\times 100$$

where *I*^956^ is the normalized intensity of the 956 cm^−1^ absorption band of amorphous PLA (PLA-f) and of the different samples. 

Color measurements were performed in a Minolta CM-3600d spectrophotometer in transmittance mode. 

Intrinsic viscosity measurements were performed at 25 °C, using an Ubbelohde viscometer and chloroform (Merck Millipore) as a solvent. Concentrations used ranged from 0.006 to 0.003 g/mL. Viscosimetric average molecular weight (*M_v_*) was calculated from the intrinsic viscosity ([*η*]) using the Mark–Houwink–Sakurada equation (Equation (2)) [28]:

(2) $$\left[\eta \right]=2.21\times 10^{-2}M_{v}{}^{0.77}.$$

Differential scanning calorimetry scans of the different samples were recorded in a TA Instruments DSC Q20 calorimeter, in nitrogen atmosphere (50 mL/min). Five milligrams of samples were weighted in standard aluminum pans and subjected to the following thermal protocol:

- A first heating scan, from 30 to 180 °C at 5 °C/min.
- An isothermal step at 180 °C for three minutes, to erase the thermal history.
- A cooling scan from 180 °C to 0 °C, at 5 °C/min.
- A second heating scan from 0 °C/min to 180 °C/min, at 5 °C/min.

The crystallinity degree of the samples was calculated using Equation (3):

(3) $$X_{c}=\frac{\Delta H_{m}-\Delta H_{cc}}{\Delta H_{\infty }}\times 100$$

where Δ*H_m_* and Δ*H_cc_* are the melting and cold crystallization enthalpies. Δ*H*_∞_ is the melting enthalpy of a perfect PLLA crystal, 93.1 J/g [17].

Thermogravimetric analysis (TGA) of the materials was conducted in a TA Instruments TGA2050 thermobalance. Ten-milligram samples were weighted in a platinum pan and heated, in nitrogen atmosphere, from 40 to 800 °C, at 10 °C/min.

Microhardness measurements were performed, six times in each sample, using a Shimadzu Type M microhardness tester. A load of 25 g, and a loading time of 10 s were used.

Films were disintegrated under simulated composting conditions at laboratory scale level following the ISO 20200 standard [29]. Film samples (15 mm × 15 mm) were buried at 4–6 cm depth in perforated plastic containers containing a solid synthetic wet waste prepared with 10% of mature compost (Mantillo, Spain), 40% sawdust, 30% rabbit food, 10% starch, 5% sugar, 4% corn oil, and 1% urea, and finally adding approximately 50 wt% of water content. The containers were incubated at aerobic conditions at 58 °C. Film samples were recovered at 4, 11, and 17 days of disintegration, washed with distilled water, dried in an oven (24 h at 37 °C), and reweighed. Photos were taken at each disintegration time and the degree of disintegrability was calculated by normalizing the sample weight at each extraction day to the initial weight. The recovered materials were also characterized by the determination of the thermal stability by means of TGA and the degree of crystallinity was determined by DSC.

## 3. Results

### 3.1. FTIR Characterization of the Samples

**Figure 2:**
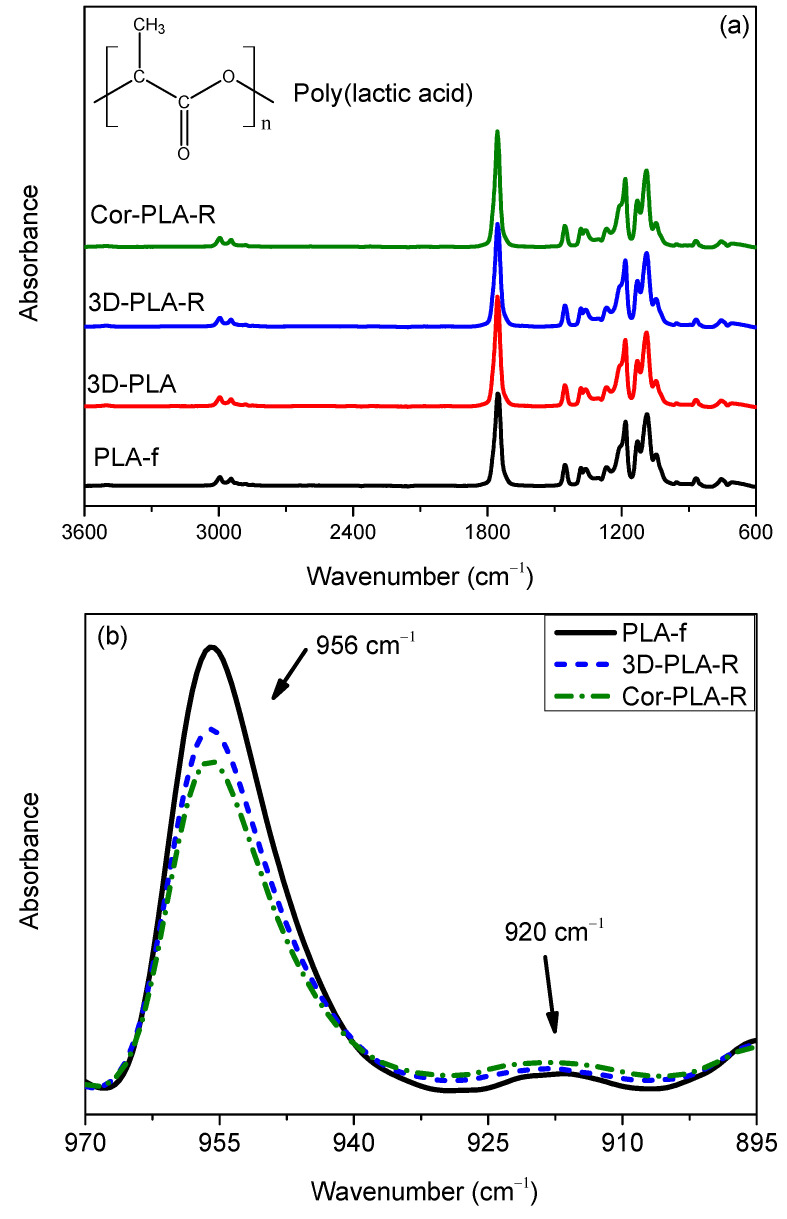
FTIR-ATR normalized spectra of the samples before and after recycling between 3600–600 cm^−1^ (**a**) and 970–895 cm^−1^ (**b**).

FTIR spectroscopy allows to study the chemical structure of the different samples. The structure of PLA and the obtained spectra are shown in Figure 2. It can be seen that all the spectra show similar absorption bands, which are characteristic in PLA materials, and are summarized in Table 2 [25]: 

The presence of these absorption bands in all the samples corroborates that all the materials are PLA. Furthermore, the fact that no important differences between the different spectra are shown suggests that both 3D printing and mechanical recycling have a limited effect on the chemical structure of PLA. However, Figure 2b shows that there are some differences in the region between 970 and 900 cm^−1^. The absorption bands centered at 956 and 920 cm^−1^ are attributed to amorphous and crystalline regions, respectively [26,27]. The decrease of the band at 956 cm^−1^, along with the slight increase of the band at 920 cm^−1^, indicate an increase of the crystallinity of the samples.

According to equation 1, the variation of the crystallinity of recycled materials with respect to amorphous PLA-f is 10% in 3D-PLA-R and 24% in Cor-PLA-R. These results show that the Cor-PLA-R sample has a higher crystallinity degree than the well-known PLA reference grade. This behavior could be due to degradation or presence of nucleating agents in the components of the 3D printing wastes mixture. For obtaining more information, FTIR-ATR spectra of each component of the mixture were recorded.

**Figure 3:**
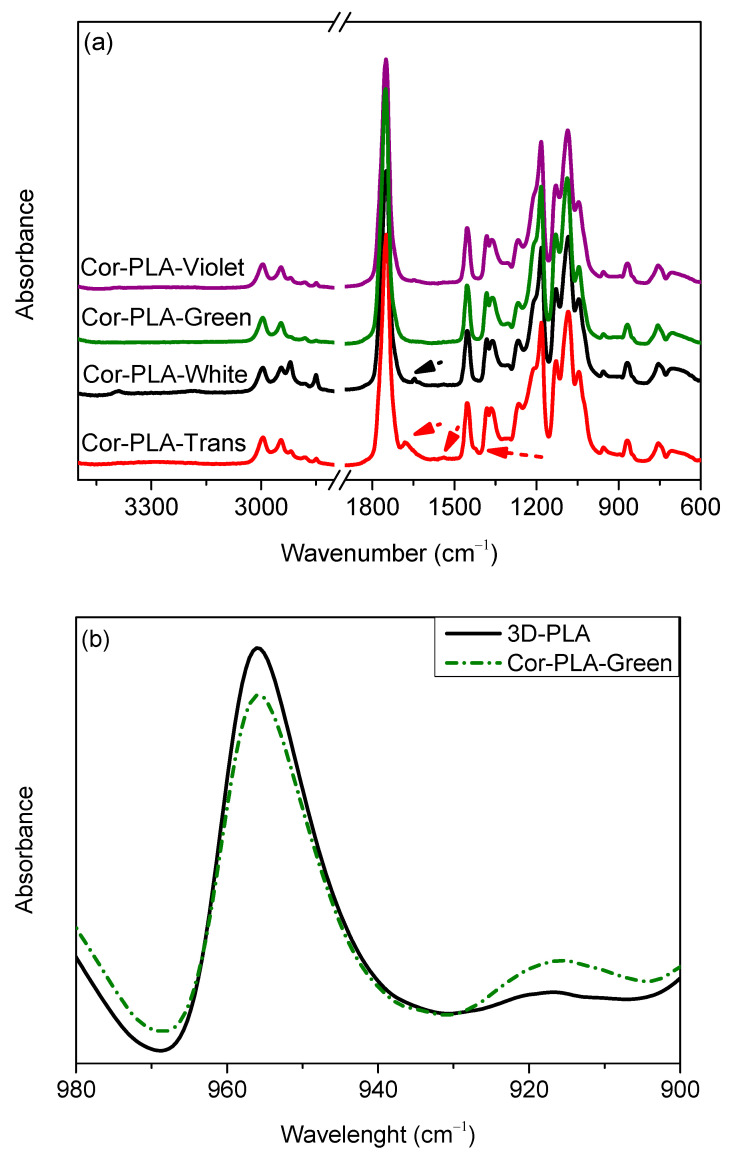
FTIR-ATR spectra (**a**) of the components of the 3D printing waste mixture, (**b**) 980–900 cm^−1^ of the FTIR spectra of 3D printing wastes.

Figure 3a shows that all the materials used for obtaining Cor-PLA-R show the characteristic PLA absorption bands. However, there are some differences between them which could affect the final properties of the blend. These differences might be attributed to the presence of different additives, which are commonly used in plastics processing field, with different objectives which includes increasing the degree of crystallinity, improving thermal and/or mechanical properties or increasing the molecular weight [3]. For instance, Figure 3a shows that the Cor-PLA-Trans and Cor-PLA-White samples present a broad absorption band over 3000 cm^−1^, and small bands at 1680 and 1540 cm^−1^. These bands could be associated to stretching of N-H bonds, amide I and amide II, respectively. These functional groups could be present in some additives used in 3D printing applications [30]. Cor-PLA-Trans also present a small band around 1420 cm^−1^, which suggest the presence of CaCO_3_ [31], which is a common additive used in plastic processing industry.

Another important difference can be observed in Figure 3b, in which it can be seen than Cor-PLA-Green has a higher absorption at 920 cm^−1^, along with a reduced absorption at 955 cm^−1^ indicating that the degree of crystallinity of the green material is higher than that of the reference material, probably due to the presence of some nucleating agent in its chemical composition. The presence of the additives, and the crystallization of some of the components of the blend should be considered, since they could also affect other properties of the recycled materials. 

### 3.2. Effect of Mechanical Recycling on the Molecular Weight of the Samples

**Figure 4:**
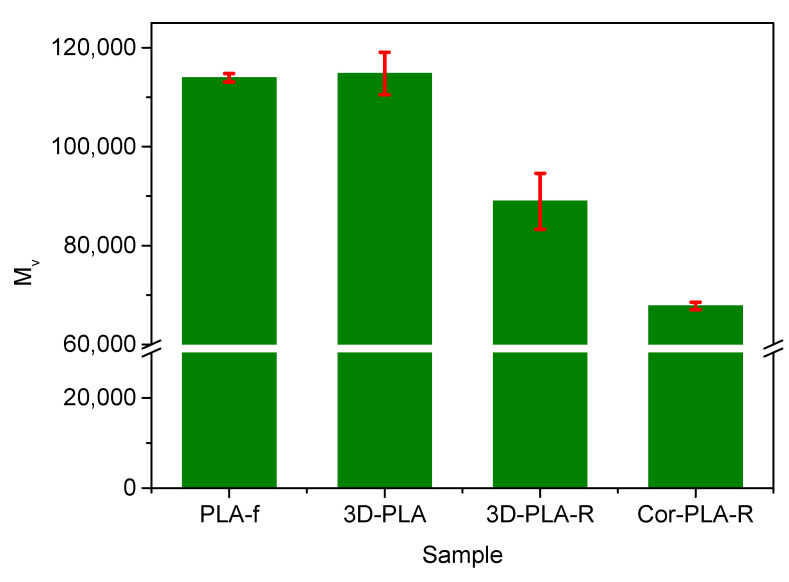
Viscosimetric average molecular weight of the samples before and after mechanical recycling.

Intrinsic viscosity, and viscosimetric average molecular weight, are fundamental parameters in polymer processing because most industrial forming processes are designed to operate in a narrow range of conditions [32]. Consequently, analyzing the effect of the mechanical recycling proves essential to assess the recyclability of PLA 3D printing wastes. Figure 4 shows the average viscosimetric molecular weight (*M_v_*) values of the different samples before and after the mechanical recycling, which were used as the main variable to evaluate the technical viability of the recycling process. It can be seen that 3D printing does not significantly affect the *M_v_* values of PLA, at least under the conditions used in this work. This behavior suggests that no important degradation reactions take place during the 3D printing process. However, the simulated washing process used at industrial recycling plants and the mechanical recycling process causes a noticeable reduction of the molecular weight of PLA, as it can be seen in the 3D-PLA and 3D-PLA-R samples. Similar results were obtained with PLA samples subjected to an accelerated ageing protocol, a washing process and melt reprocessing [17]. During service, washing and reprocessing steps, PLA is subjected to several degrading agents, such as temperature and water. The degradation mechanism involved in the thermomechanical degradation of PLA might include hydrolysis, oxidative degradation, backbiting reactions, and intermolecular transesterification reactions [17,33]. 

**Figure 5:**
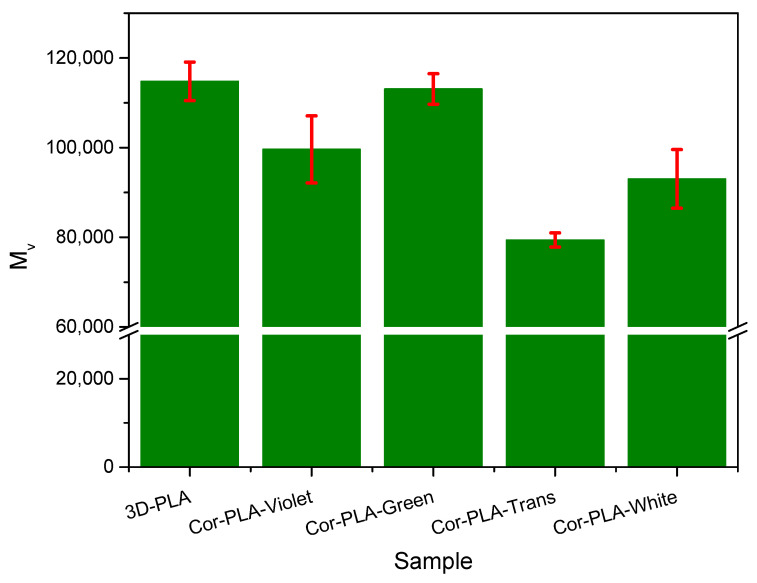
Viscosimetric average molecular weight of the components of the 3D printing wastes.

Although both recycled materials show lower *M_v_* values than the 3D-PLA sample, the molecular weight is lower in the 3D-blend based on different PLA wastes coming from the coronamaker agents. In order to better understand this behavior, the *M_v_* values of the different components of the mixture of 3D printing wastes prior to the washing and reprocessing steps are shown in Figure 5. It can be seen that from the components of the blend, only the green-colored PLA 3D printing waste has a molecular weight similar to that of the reference grade, while the other components show significantly lower values. These low *M_v_* values of some of the components of the waste mixture are responsible for the average molecular weight of the Cor-PLA-R blend sample and could be attributed to the presence of different PLA grades, and/or different processing conditions of each individual sample. This behavior highlights that the heterogeneity of the waste streams is one of the biggest challenges for mechanical recycling of plastic waste. A potential alternative is the implantation of distributed recycling scheme, in which the adequate selection of a PLA 3D printing wastes stream plays a prominent role. In fact, materials such as the 3D-PLA or the Cor-PLA-Green samples could lead to recycled materials with interesting properties. 

### 3.3. Thermal Properties of the Recycled Materials Samples

**Figure 6:**
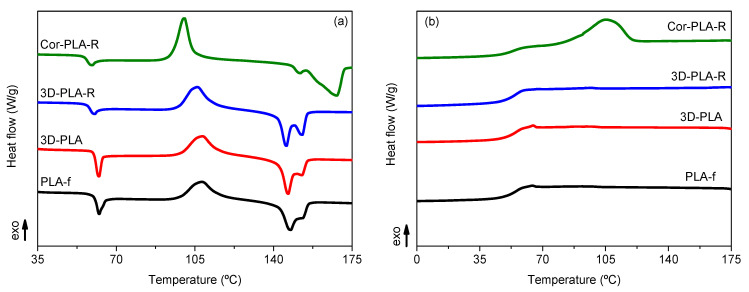
Differential scanning calorimetry (DSC) first heating (**a**) and cooling (**b**) scans of the samples before and after mechanical recycling.

Intrinsic viscosity results showed that mechanical recycling led to the degradation of PLA. Furthermore, the heterogeneity of the PLA 3D printing wastes blend could affect the final properties of the recycled materials. Therefore, the thermal properties of the different samples were studied by means of DSC and TGA tests. Figure 6 and Table 3 shows the DSC results of the different recycled materials. 

Overall, Figure 6a shows that the different samples present the characteristic thermal behavior of PLA. Firstly, a glass transition around 60 °C, accompanied by an endothermic peak which is related to relaxation enthalpy characteristic of the physical ageing of PLA [34,35]. Secondly, there is an exothermic peak above 100 °C, assigned to the cold crystallization temperature of PLA. Lastly, there is an endothermic double peak, assigned to the melting of the polymer. It can be seen in both Figure 6a and Table 3 that the samples coming from the reference grade show a similar behavior, although, a small decrease of the cold crystallization temperature in the 3D-PLA-R sample can be observed. Such decrease can be attributed to the degradation of PLA during the washing and reprocessing step, since shorter polymer chains crystallize more easily. Similar results have been observed in a commercial grade of PLA subjected to an ageing protocol and similar washing and reprocessing conditions [36] and during repeated 3D printing cycles [11]. 

Another important parameter for printed pieces is the crystallinity degree. Table 3 shows that there are no important differences between the materials coming from the well-known reference grade, although a slight crystallization was observed by means of FTIR spectroscopy. Nevertheless, the differences are small and could be attributed to the different sensibility of both techniques. 

**Figure 7:**
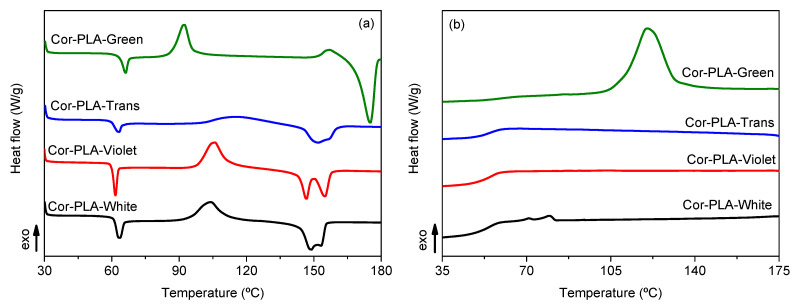
DSC first heating (**a**) and cooling (**b**) scans of the components of the 3D printing waste.

Regarding the recycled mixture of the 3D printing wastes, Table 3 and Figure 6 show that the Cor-PLA-R sample presents important differences when compared with the reference grade samples. A noticeable decrease of the cold crystallization temperature can be seen on Figure 6a and Table 3, which could be related to the presence of shorter polymer chains, as it was observed in the viscosimetric average molecular weight results, or by the presence of nucleating additives, as it was seen by FTIR-ATR spectroscopy. The increased crystallization ability of the Cor-PLA-R sample can also be observed in Figure 6b, since the recycled mixture of 3D printing wastes is the only sample which shows a crystallization peak during the DSC cooling scan. Another important difference between the Cor-PLA-R and the reference grade samples can be observed in the melting behavior. The recycled PLA blend shows a significantly higher melting temperature, which does not agree with the degradation observed by means of solution viscometry, since shorter polymer chains form less perfect crystals that melt at lower temperatures. Nevertheless, it should be mentioned that the presence of nucleating agents could lead to the formation of more perfect crystalline structures, which melt at higher temperatures. To better understand this behavior, the first heating and cooling DSC scans of the components of the 3D printing waste blend are shown in Figure 7. 

Figure 7 shows that, despite all the materials being PLA, there are important differences in the thermal behavior of the components of the 3D printing wastes blend. It can be seen that the white (Cor-PLA-White) and violet (Cor-PLA-Violet) samples show a similar thermal behavior, while the translucent (Cor-PLA-Trans) and green (Cor-PLA-Green) samples show some differences. In the case of the Cor-PLA-Trans sample, both the cold crystallization and melting peaks are very small, indicating that this PLA grade has a low crystallization ability. Regarding the Cor-PLA-Green, it can be seen on Figure 7a that it shows a lower *T_cc_* value and a significantly higher *T_m_* in comparison with the other samples. Furthermore, Figure 7b shows that the green sample is the only material that presents a crystallization peak during the cooling scans. These results point out that the Cor-PLA-Green sample is the responsible of the increased crystallization ability and higher *T_m_* values of the Cor-PLA-R blend sample. This increased crystallization ability was also observed by means of FTIR-ATR spectroscopy, and can be related to the presence of a nucleating agent in the Cor-PLA-Green sample.

**Figure 8:**
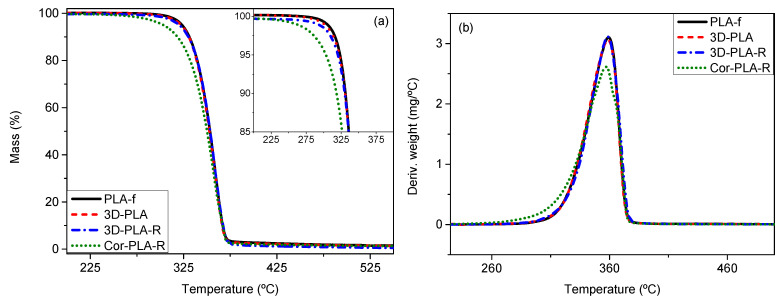
(**a**) TGA curves and (**b**) derivative thermogravimetric analysis (TGA) curves of the samples before and after mechanical recycling.

The thermal stability of the materials is also an important property from the processing point of view. TGA curves of the PLA reference grade and the recycled materials are shown on Figure 8a. All the films showed a complete weight loss in a single degradation step, with a maximum degradation temperature in the range of 250–400 °C (Figure 8b) confirming again that the polymeric matrix is PLA. It can be seen that the TGA curves of the samples coming from the reference grade are very similar, only showing a slight decrease of the thermal stability after 3D printing and recycling, probably related to the degradation of the polymer during printing and recycling process [11,17]. However, it can be seen that the Cor-PLA-R sample has a significantly lower thermal stability than the other samples. This behavior is in good agreement with those of the viscosimetric average molecular weight, since the shorter polymer chains degrade at lower temperatures [37]. These results highlight that the heterogeneity of the wastes plays a very important role on the final properties of the recycled materials, posing an important technical barrier for the recycling of 3D printing wastes. Distributed recycling could help to mitigate this problem, since it allows to recycle carefully selected waste streams, consisting of only one polymer grade with well-known properties.

### 3.4. Vickers Hardness of the Recycled Materials

**Figure 9:**
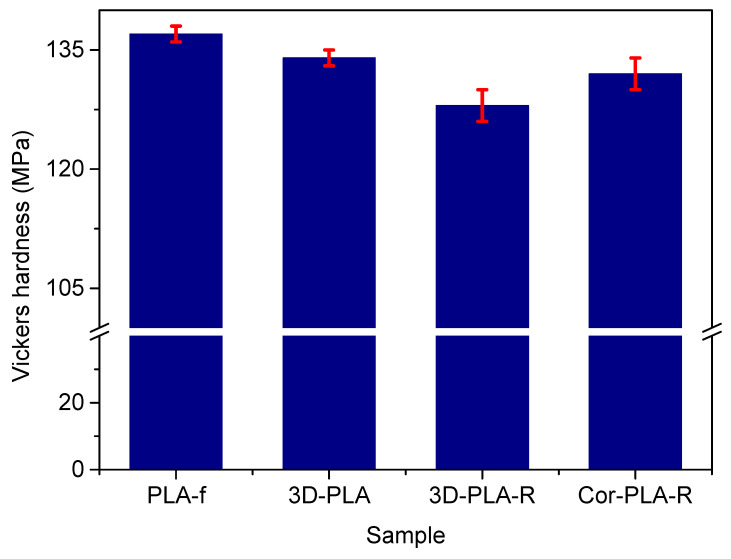
Vickers hardness values of the samples before and after mechanical recycling.

Mechanical properties play a very important role on film samples, since they could dictate the applications in which the final products will be used. Microhardness measurements were conducted to analyze the effect of mechanical recycling process on the Vickers hardness of the different materials, and the obtained results are shown on Figure 9. It can be seen that, although differences are small, 3D printing and mechanical recycling led to a decrease of the Vickers hardness of the reference PLA grade film. This slight reduction of the hardness could be related to the degradation of the polymer observed in the viscosimetric average molecular weight results. Nevertheless, previous studies conducted by Pillin et al. [38], Perego et al. [39], and Agüero et al. [8] pointed out that the effect of a decrease on the molecular weight of PLA on its hardness is limited.

Figure 9 also shows that the PLA-based blend resulting from the mixture of the 3D printing PLA wastes have a slightly higher hardness value than the recycled reference grade, despite the significantly lower *M_v_* values observed. However, the higher surface crystallinity of Cor-PLA-R, observed by FTIR-ATR spectroscopy and DSC, could result in an increased hardness value. These results point out that films based on recycled 3D materials show good mechanical properties, especially the blend based on a mixture of 3D printing wastes, which could be an important factor in certain applications.

### 3.5. Optical Properties of the Recycled Materials

**Figure 10:**
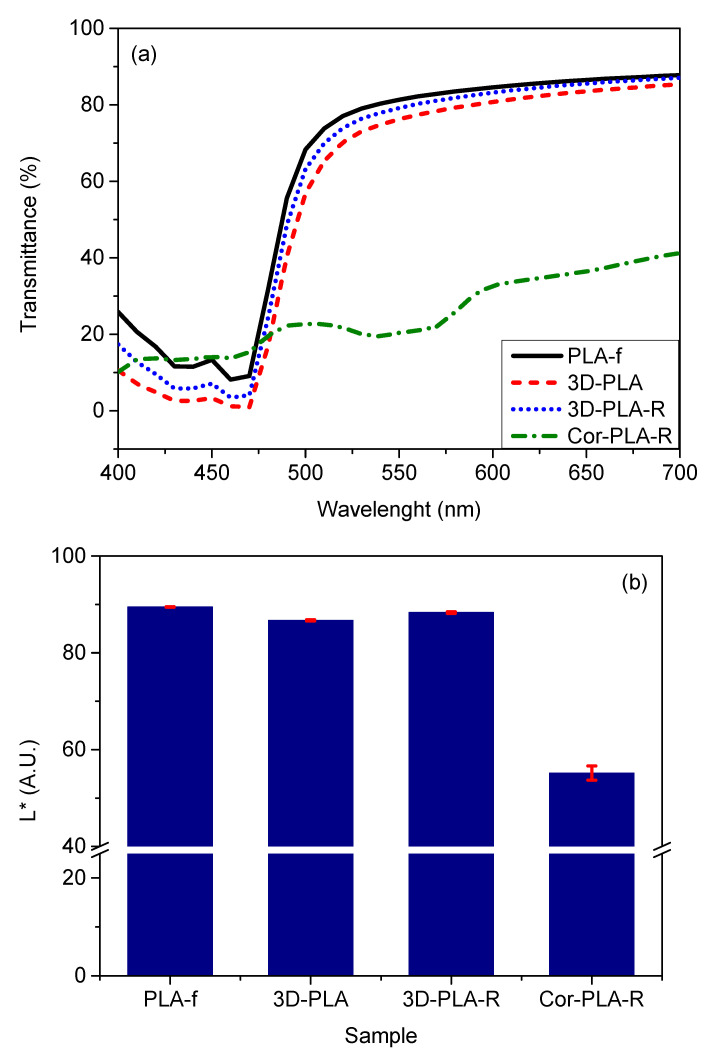

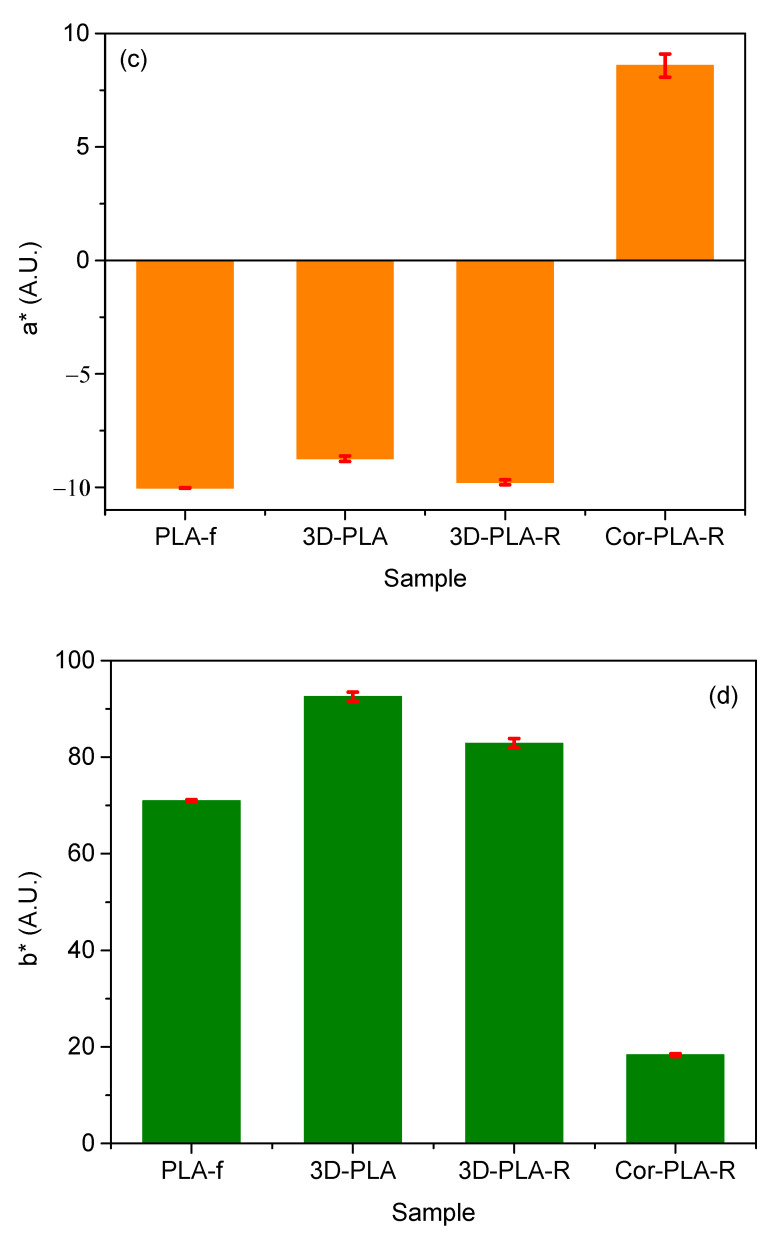
Visible spectra (**a**) and L* (**b**), a* (**c**), and b* (**d**) parameters of the different samples.

**Figure 11:**
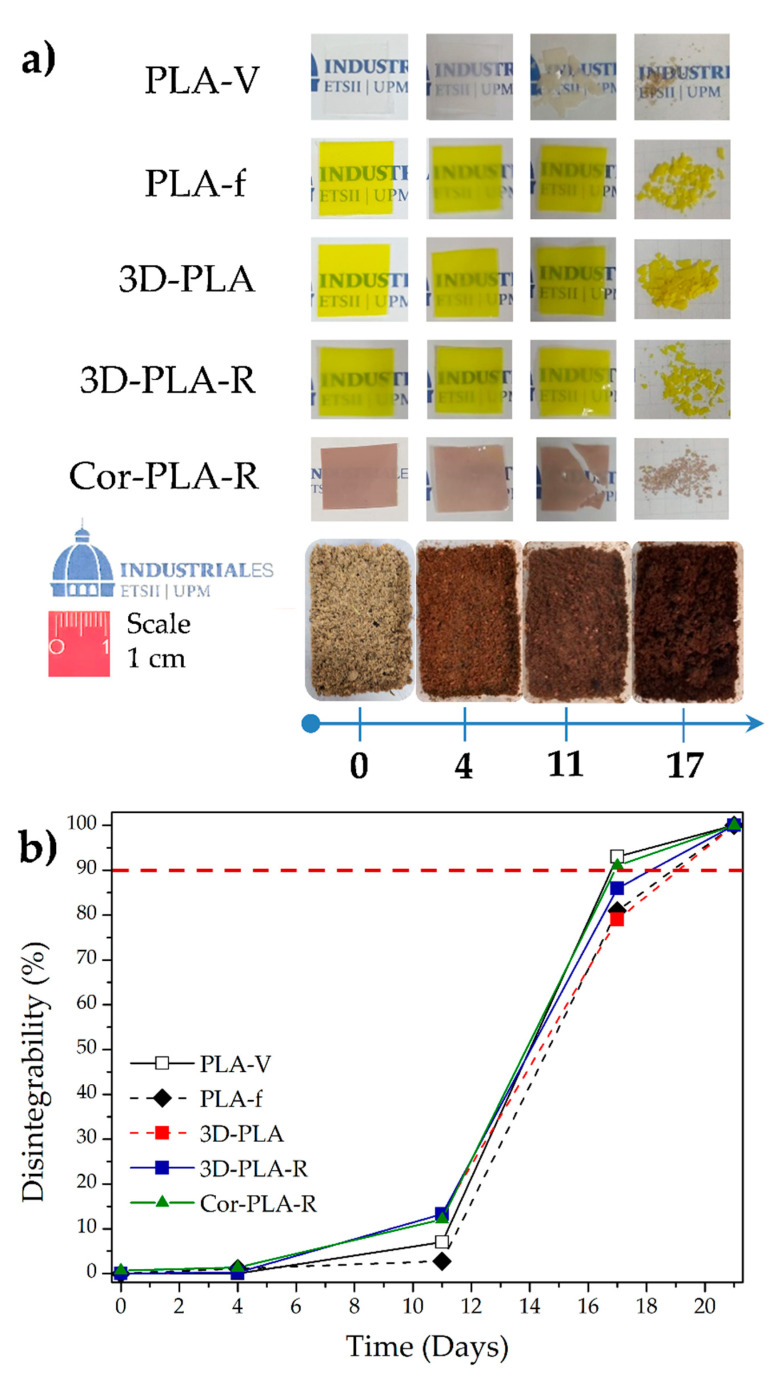
(**a**) Visual appearance of films samples before and after exposition at compost conditions, (**b**) films disintegration degree under composting conditions as a function of time and compost evolution during the composting test.

The optical properties of films play a very important role in several applications, particularly when seeing through the film is desired. In fact, PLA is widely accepted as film for several industrial sectors due to its high transparency. Consequently, colorimetric measurements were performed to analyze the effect of the mechanical recycling of the different wastes. Figure 10 shows the spectra and the L* a* b* coordinates of the different materials. Meanwhile, the visual appearance of the samples can be seen on Figure 11a, along with a neat and recycled PLA obtained under controlled conditions (PLA-V and PLA-R) [17]. It can be seen that, overall, the samples coming from the well-known reference grade show very similar optical properties. PLA-f, 3D-PLA, and 3D-PLA-R show high lightness values, indicating that these samples have a low light absorption. Furthermore, all the samples coming from the reference grade show a strong absorption around 470 nm, and high b* values, which correspond to the strong yellow color observed in these samples (Figure 11a).

Regarding the Cor-PLA-R sample, Figure 11a show that its visual appearance is very different from that of the reference grade. Meanwhile, the spectra of Cor-PLA-R shows a broad absorption band between 500 and 600 nm (Figure 10a). Moreover, the a* (Figure 10c) and b* (Figure 10d) values are under 20, resulting in the purplish gray color observed in this sample. This behavior is a consequence of the presence of different colored PLA wastes in the blend. These results point out that the mechanically recycled 3D printing wastes coming from a reference grade show visual properties close to those of the virgin material. However, the recycled blend coming from the mixture of PLA 3D printing wastes could be destined to applications in which the visual properties are not so important.

### 3.6. Disintegration under Composting Conditions

As it was already commented, PLA cannot be recycled too many times since it experiences a reduction on the molecular weight, which finally leads to a reduction of overall thermomechanical properties. Thus, the disintegrability behavior under composting conditions of the films was evaluated at laboratory scale level, with the main objective to evaluate composting as end-life option for recycled PLA materials after their useful life. Neat PLA was also assessed for comparison. The visual appearance of the films (Figure 11a) shows that neat PLA (PLA-V) became opaque after the fourth day of disintegration, although the material practically did not change its initial mass. The changes on the PLA transparency during disintegration has been ascribed to a change in the refraction index, as a consequence of water absorption and the formation of shorter polymeric chains during the hydrolytic degradation process. This suggests that the disintegration process of PLA matrix has started [9,40,41]. This behavior was less evident in the case of recycled materials based on 3D printing wastes, probably due to these films being colored, making it difficult to observe those changes. After 11 days of incubation under composting medium, PLA-V and Cor-PLA-R films became brittle. Furthermore, recycled materials show a higher mass loss, probably due to the shorter polymer chains generated during the recycling process. These short chains further plasticize the polymeric matrix, becoming less brittle and at the same time being more susceptible to migrate to the compost medium. It is also worth to note that the increased polymer chain mobility of recycled materials helps the diffusion of water into the polymeric matrix, resulting in greater hydrolysis which in turn results in the generation of short-chain oligomers and/or small molecules that are susceptible to microorganisms enzymatic degradation [42,43]. After 17 days of the composting disintegration test, small pieces of films were recovered. As expected, among the films based on 3D-printed materials, the recycled ones reach firstly the 90% disintegration, which is frequently used as the goal of the disintegrability test [40,43]. Finally, after 21 days, the materials completely disappeared. The success of the test was also followed by the determination of the pH at the end of the test (pH around 7) and by following the evolution of the compost soil which finally resulted in humus-rich soil (Figure 11b). 

The thermogravimetric analysis showed the changes on the thermal stability during the exposition to the composting medium. The onset degradation temperatures (T_5_) and maximum degradation temperatures (*T_max_*) values are summarized in Table 4. Both thermal parameters decreased with increasing composting time. The decrease in T_5_ value after 4 days under composting was more marked than that after 11 days, which could be ascribed to the loss of short polyester chains (caused by hydrolysis during the initial disintegration steps), which are susceptible to enzymatic degradation [44]. Finally, practically no residue was observed at 800 °C (between 0.5 to 2.2%), suggesting that the inorganic composition is too low in the starting 3D-PLA wastes.

The degree of crystallinity was calculated from the DSC first heating scan before and after 4 days under composting and the results are also summarized in Table 4. As it was already commented films presented low crystallinity before the disintegration in compost. However, after 4 days in contact with the compost medium, the degree of crystallinity significantly increased due to the presence of shorter polymer chains formed owing to the hydrolysis process that are able to crystallize easily. This behavior was particularly marked in Cor-PLA-R film since it is the material which initially has higher amounts of short polymer chains as it was determined by the solution viscometry measurements.

### 3.7. Solid-State Polymerization (SSP) of Recycled Materials

As it has been pointed out in previous sections, mechanical recycling causes a decrease on important properties and on the viscosimetric average molecular weight of the recycled materials, which could affect their printability, and ultimately have a negative impact on the demand of recycled materials for 3D printing applications. To improve the properties of the recycled materials, a thermal treatment was applied to the samples to promote the SSP of PLA, and thus increase the molecular weight of the materials. SSP consists in heating a polyester, such as PLA or PET, at temperatures between *T_g_* and *T_m_*. At this range of temperature, polycondensation reactions are promoted in the amorphous region of the polymer, while undesirable byproducts, such as water, are removed by continuous vacuum conditions. However, the time and temperature conditions should be carefully chosen, since both degradation and polycondensation reactions could take place [45,46]. This behavior is due to the two thermodynamic equilibria that govern the polycondensation process to obtain PLA: (i) Hydrolysis/condensation for ester formation and (ii) ring/chain equilibrium for the formation of lactide [47].

This method could be really useful since it does not require special equipment nor additional additives. Hence, they could be available for a large number of manufacturers and processors. Nevertheless, special attention should be paid on the polymerization process, since it is affected by the diffusion of the reactive end groups inside the polymer, and by the diffusivity of the byproducts from the polymer particles [45,46,47,48,49].

**Figure 12:**
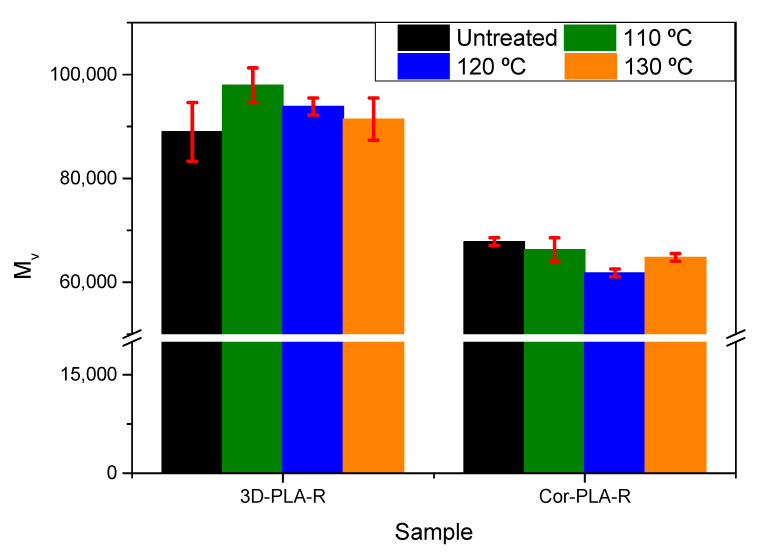
Viscosimetric average molecular weight of the samples after SSP.

Figure 12 shows the viscosimetric molecular weight values and it is possible to observe that the effect of the SSP process varies with the kind of sample used. On the one hand, it can be seen that for the 3D-PLA-R sample, a slight increase of the Mv values was achieved after the SSP treatments, especially at 110 °C. On the other hand, the SSP had no positive effects on the Cor-PLA-R samples. This behavior might result surprising, since the lower the molecular weight, the more effective the SSP process should be [45]. However, DSC scans showed that Cor-PLA-R has an increased crystallization ability. This high crystallization ability of the Cor-PLA-R sample might be resulting in a decreased efficiency of the SSP process due to the lack of mobility of the polymeric chain-ends and the reduced diffusivity of the byproducts of the reaction. According to Peng et al. [50], crystallization could decrease the byproduct diffusivity and chain-end mobility, limiting the effectiveness of the SSP process. 

These results suggest that, from a properties point of view, obtaining a mechanically recycled PLA, based on 3D printing wastes, with acceptable properties is feasible. However, is it better to use a well-known reference grade, since the presence of different PLA grades in the Coronamakers waste blend led to some important differences in the properties of the recycled plastic. This could be possible in a distributed recycling scheme, in which each manufacturer recycles its own known 3D printing wastes. Furthermore, it is possible to increase the viscosimetric average molecular weight of the well-known reference grade by SSP, although, conditions should be optimized in order to further increase the average molecular weight of degraded wastes. 

## 4. Conclusions

In this study the mechanical recycling of different PLA-based 3D printing wastes was evaluated, along with proposing a cost-friendly and environmentally sound method to improve the properties of the recycled materials. Two different kind of 3D printing wastes were considered, (i) a well-known reference grade, and (ii) a mixture of wastes kindly supplied coronamakers agents in Madrid. The wastes were subjected to a demanding washing process to simulate the washing conditions used at industrial level during recycling process, and then melt reprocessed by extrusion followed by compression molding to obtain films. The obtained materials were characterized with the aim to evaluate the possibility to recover those waste into useful films for several applications. Intrinsic viscosity values were used to calculate the viscosimetric molecular weight and the results point out that the mechanical recycling process led to the degradation of PLA, although, the effect was more severe in the blend of printing wastes. It is worth to note that the samples coming from the reference grade showed only small variations on the thermal, mechanical and optical properties. However, the blend prepared from the mixture of 3D printing wastes had an increased crystallization ability, which was attributed to one of the components of the blend. Furthermore, the visual aspect of the recycled materials was significantly affected in the case of the recycled waste blend, suggesting that applications in which good aesthetic properties are needed might be unsuitable for this kind of material. All the recycled materials were successfully disintegrated under composting conditions in 21 days, showing their overall sustainable character as they can close the loop of the circular economy. The films based on 3D-recycled wastes showed somewhat higher disintegration kinetic than the neat PLA filament due to the shorter polymer chains are more susceptible to hydrolysis and microorganism’s attack. Finally, the recycled materials were subjected to an SSP process at different temperatures (110, 120, and 130 °C) as another way to revalue 3D-waste residues. SSP led to a slight increase of the average viscosity molecular weight of the recycled well-known reference grade, especially at 110 °C. However, no improvements were observed in the blend based on 3D printing waste mixture. 

The obtained results suggest that the implantation of a distributed recycling scheme, which allows to recycle 3D printing wastes consisting in a single and well-known PLA grade, could lead to the mechanically recycled materials with good performance, which are compostable after their useful life. It was also possible to increase the average molecular weight of these recycled materials, although further optimization of the SSP conditions is needed in order to improve the properties of severely degraded materials. These results lead to increased circularity of the 3-D printing processes with PLA and might allow researchers and technicians to lay the groundwork for the development of better distributed recycling schemes.

## Figures and Tables

**Table 1:** Description of the materials used in this study.

Sample	Description
PLA-V	Reference PLA grade, processed under controlled conditions [17]
PLA-f	Smartfil^®^ PLA filament
3D-PLA	PLA-f printed in a ABAX PRI5 3D printer at 200 °C.
3D-PLA-R	Mechanically recycled PLA-f printing wastes
Cor-PLA-R	Mechanically recycled blend of the coronamakers association printing wastes
Cor-PLA-green	Green-colored coronamakers association printing wastes
Cor-PLA-violet	Violet-colored coronamakers association printing wastes
Cor-PLA-white	White-colored coronamakers association printing wastes
Cor-PLA-Trans	Translucent coronamakers association printing wastes
3D-PLA-R-TXXX	3D-PLA-R sample subjected to a thermal treatment, in which XXX is the temperature (110, 120 or 130 °C).
Cor-PLA-R-TXXX	Cor-PLA-R sample subjected to a thermal treatment, in which XXX is the temperature (110, 120 or 130 °C).

**Table 2:** FTIR-ATR bands assignation.

Wavenumber (cm^−1^)	Assignation
2995	ν_as_ CH_3_
2945	ν_s_ CH_3_
1755	ν C=O
1451	δ_as_ CH_3_
1383	δ_s_ CH_3_
1360	δ_1_ CH
1268	δ CH_3_ and ν COC
1212–1185	ν_as_ COC and r_as_ CH_3_
1130	r_as_ CH_3_
1090	ν_s_ COC
1045	ν C-CH_3_
956–920	r CH_3_ and ν CC
868	ν C-COO

**Table 3:** DSC first heating scan results of the different samples.

Sample	T_cc_ (°C)	T_m_ (°C)	ΔH_cc_ (°C)	ΔH_m_ (°C)	*X_c_* (%)
PLA-f	108.1	147.7–153.2	22.3	23.4	1
3D-PLA	108.3	146.5–152.9	21.2	23.1	2
3D-PLA-R	106.1	145.8–152.8	25.5	26.2	1
Cor-PLA-R	100.3	151.9–168.2	28.5	32.2	4

**Table 4:** TGA thermal parameters and degree of crystallinity determined by DSC for all films at different disintegration times.

Film Sample	Disintegration Time (Days)	T_5%_ (°C)	T_max_ (°C)	Residue at 800 °C (%)	*Xc* (%)
PLA-V	0	325	366	1.5	2
	4	270	319	1.3	35
	11	245	339	1.3	-
PLA-f	0	323	359	1.5	1
	4	288	338	0.8	24
	11	257	348	1.1	-
3D-PLA	0	323	359	1.5	2
	4	285	332	2.2	24
	11	249	340	0.6	-
3D-PLA-R	0	321	359	1.5	1
	4	285	331	1.3	25
	11	248	344	0.6	-
Cor-PLA-R	0	303	357	1.5	4
	4	260	332	1.7	37
	11	250	343	1.4	-

## Data Availability

The data presented in this study are available on request from the corresponding author. The data are not publicly available due to restrictions.
